# A temperature prediction model for aluminum smelting furnaces considering dynamic temporal and spatial features

**DOI:** 10.1038/s41598-026-45466-5

**Published:** 2026-04-07

**Authors:** Jiayang Dai, Lei Wang, Shenwang Li, Thomas Wu, Zhuhua Chen, Zhen Chen

**Affiliations:** 1 Guangxi Key Laboratory of Intelligent Control and Maintenance of Power Equipment, School of Electrical Engineering, Guangxi University, Nanning 530004 Guangxi, China; 2 State Key Laboratory of Featured Metal Materials and Life-cycle Safety for Composite Structures, Guangxi University, Nanning 530004 Guangxi, China

**Keywords:** Aluminum smelting furnace, Temperature prediction, Graph convolutional network, Dynamic graph fusion structures, Long short term memory network, Energy science and technology, Engineering, Materials science, Mathematics and computing

## Abstract

Aluminum smelting relies on precise furnace temperature measurement to ensure product quality and yield, yet accurate real-time measurement faces technical barriers: thermocouples are susceptible to damage in high-temperature environments, resulting in increased measurement costs, while protective sheaths of thermocouples introduce thermal inertia, causing delayed temperature response in molten metal monitoring. Furnace temperature prediction models are usually used to achieve accurate prediction of furnace temperature to solve these issues. However, traditional methods often struggle to manage the dynamic spatial-temporal relationships among variables, leading to suboptimal predictive outcomes. Considering the aforementioned challenges, a proposal of a furnace temperature prediction model is made. The proposed model utilizes a graph convolutional network that combines dynamic graph fusion structures to capture evolving spatial correlations, supplemented by a sliding fully connected LSTM architecture with gating mechanisms, aiming to extract temporal dependencies through time series data analysis. Experimental results obtained from real-world aluminum smelting furnace data demonstrate that the proposed model achieves superior prediction accuracy compared to LSTM and Transformer baselines. Ablation experiments further validate the critical contributions of each architectural component. These results validate its ability to manage significant numerical variations and dynamic spatial-temporal relationships, establishing it as a robust solution for real-time temperature prediction in aluminum smelting furnaces.

## Introduction

Aluminum is one of the most produced non-ferrous metals across the globe, which is extensively applied in numerous sectors, including new energy vehicles, power transmission, aerospace, and packaging. The reasons behind its extensive application lie in its low density, excellent electrical and thermal conductivity, and good corrosion resistance. The temperature of the smelting furnace significantly influences both aluminum yield and product quality during industrial production processes. However, real - time and accurate furnace temperature measurement at industrial sites is often difficult. Thermocouples are susceptible to damage in high-temperature environments, which raises measurement costs. Also, the protective sheaths of thermocouples create thermal inertia, leading to a delayed temperature response when monitoring molten metal. Therefore, a robust furnace temperature prediction framework must be established to enable industrial-grade real-time process monitoring, thereby addressing these technical barriers and guaranteeing consistent high-quality aluminum production.

To overcome these hardware limitations, the industry has increasingly adopted temperature prediction. Temperature prediction models for aluminum smelting furnaces are broadly classified into two categories: mechanistic modeling^1–3^ and data-driven modeling^4,5^. Mechanistic modeling relies on physical and chemical principles and is capable of effectively describing operational states. Many studies aim to optimize industrial furnace operations through mechanistic modeling. Wang et al.^6^ developed a 3D model for an aluminum melting furnace, incorporating melting, burner inversion, and combustion capacity, and used FLUENT UDF and hybrid programming to explore burner effects on melting. Tudón-Martínez et al.^7^introduced a 0D model to optimize industrial box furnaces, simplifying complex processes by aggregating energy conservation equations and considering key design parameters. Both models simplify complex physical processes to reduce computational complexity and provide a basis for improving energy efficiency and operational parameters. Mechanistic modeling yields accurate predictions when the underlying physicochemical processes are sufficiently understood to permit closed-form or low-order differential descriptions. It offers a robust scientific basis for system analysis and decision-making. However, aluminum smelting involves complex, multi-scale physical and chemical interactions, such as electrochemical reactions and multiphase flows. These intricate processes make it computationally challenging to develop comprehensive mechanistic models, especially given the substantial resource demands they impose across various industrial settings^6,8^. In contrast, data-driven modeling eliminates the need for exhaustive investigation and simulation of intricate industrial mechanisms. Instead, it only requires a basic understanding of the fundamental principles governing the system under study. Models can be constructed using target data and associated variables, making this approach highly advantageous for simulating complex industrial processes. Nevertheless, the accuracy of data-driven models heavily depends on the quality of the input data. While data-driven modeling offers increased flexibility and lower computational requirements compared to mechanistic approaches, it is crucial to rigorously validate the quality and representativeness of the data to ensure accurate reflection of the system’s operational dynamics^9–11^. The combination of mechanism-based and data-driven methodologies requires a context-aware selection of modeling strategies, emphasizing application-specific operational constraints and performance targets.

Previous data-driven models, such as Artificial Neural Networks (ANNs) and Recurrent Neural Networks (RNNs)^12–14^, have encountered difficulties in capturing long-term dependencies within yield data. Additionally, these models often suffer from gradient explosion or vanishing. The Long Short-Term Memory (LSTM)^15^ architecture was proposed to resolve gradient-related limitations in sequential modeling. This innovation introduces gated memory cells that adaptively maintain crucial temporal dependencies over long periods and discard outdated information via conditional forgetting. The LSTM architecture specifically regulates information flow through three gating mechanisms: an input gate governing feature incorporation into the cell state, a forget gate determining obsolete information elimination, and an output gate controlling state-dependent feature exposure. These gates collectively enable adaptive long-term dependency learning through dynamic memory management. LSTM-based architectures demonstrate superior capability in capturing long-term temporal dependencies, leading to their widespread adoption across diverse sequential prediction tasks. Kun et al.^16^ developed an attention-enhanced LSTM model for air temperature prediction, demonstrating superior robustness and accuracy compared to the standard LSTM architecture. R. Jin et al.^17^. proposed a Bi-LSTM two-stream network for machine RUL prediction. It processes multi-source data with dual Bi-LSTM branches, fuses features for estimation, and achieves the best results on benchmark datasets. This approach offers a robust solution for predictive maintenance. However, when modeling datasets characterized by rapidly fluctuating values, it is challenging for these models to capture the evolving patterns within the dataset. Consequently, achieving precise modeling is difficult, and most models exhibit a significant delay in response. In contrast to LSTM, the Transformer^18^ utilizes self-attention to directly model relationships between any two positions within a sequence. By processing the entire sequence in parallel rather than sequentially, the Transformer is capable of efficiently capturing global dependencies. Zhou et al.^19^ proposed the Informer, a Transformer-based model for long sequence time series forecasting. It uses ProbSparse self-attention and a non-iterative decoder, achieving a 20% MSE reduction and 5x faster training than traditional Transformers. This makes it suitable for applications like smart grid demand forecasting. Liang et al.^20^ proposed CrossFormer for multivariate time series forecasting. By using cross-dimensional attention, it captures inter-variable and temporal patterns. Evaluated on traffic and energy datasets, it reduces MAE by 15.3% and speeds up training by 30% compared to Informer, showing strong dependency modeling for industrial and smart city applications. However, the Transformer model features a sophisticated architecture, incorporating a substantial quantity of parameters, requiring large-scale datasets for effective training to attain optimal performance. Additionally, it necessitates considerable computational resources, particularly when processing large-scale datasets.

However, the aforementioned architectures—whether recurrent or self-attention-based—are designed on the assumption of a fully-connected or grid-like topology, and thus ignore the non-Euclidean spatial relationships that widely exist in industrial sensor networks. Recent advances in spatiotemporal modeling have highlighted the critical role of spatial dependencies. Graph Convolutional Networks (GCNs)^21–23^ effectively capture these structural relationships by aggregating features through graph topology and node attribute integration. By using the correlation between variables, we can construct the topological relationship between variables. To extract spatial features from process variables, Zanfei et al.^24^ developed a hybrid framework combining RNNs for temporal feature extraction with GCNs for graph-structured dependency encoding in multi-sensor water demand prediction. This spatial-temporal integration enhanced predictive accuracy under sensor malfunction scenarios by explicitly modeling missing data resilience through topological relationships. D. Luo et al.^25^. established a unique framework for dissolved gas level prediction in transformer oil through Temporal Convolutional Network (TCN) and GCN integration. TCN extracts features from gas data over time. GCN then predicts future gas levels via a graph structure that maps gas relationships, with a matrix based on correlation coefficients. Despite notable advancements in prior research, a critical limitation hindering GCN’s performance persists within existing architectures. Traditional GCNs utilize static graphs to represent the interrelationships among variables. However, industrial processes exhibit dynamic operational modes, resulting in time-varying interdependencies. Static graphs are incapable of accurately capturing these evolving relationships. Consequently, the exploration of dynamic graphs to depict the varying connections is imperative for the precise extraction of spatial features^26,27^.

In order to tackle the above challenges, a novel deep learning model: A network that combines dynamic graph convolutional networks and sliding fully-connected LSTM is proposed. The model is capable of processing variable data on the global graph, which is based on Grey Relation Analysis (GRA) of variables, and effectively capturing dynamic spatial-temporal features in real time. The principal contributions of this research are as follows:

- A dynamic graph fusion mechanism is developed to determine the dynamic spatial correlations of features. The study encompassed the dynamic correlations between features across various time steps, as well as the dynamic similarities in the numerical transformations of these features over time. Subsequently, these dynamic graphs were integrated with the initial graph structure. Utilizing the attention mechanism, the learning process was refined to identify the dynamic spatial correlations of features.
- A sliding fully-connected LSTM network is designed to analyze the temporal dependence of variables, which consists of several fully-connected LSTM units for extracting temporal features from the original time series. The sliding network is designed to capture rapid changes in data trends.
- Comprehensive experiments are carried out on real aluminum smelting furnace datasets, which substantiate that our model attains superior predictive performance in comparison to the extant models.

The paper is structured as follows: Section "Investigation of challenges in aluminum smelting process" characterizes the aluminum smelting process in regenerative furnaces and analyzes operational challenges. Section “Methodology” proposes the DYGCN-SLSTM soft sensor framework. Section “Experiments” validates the model’s effectiveness through industrial case studies using production data from operational smelting plants. Section “Conclusion” concludes with technical insights and implementation recommendations.

## Investigation of challenges in aluminum smelting process

**Fig. 1:**
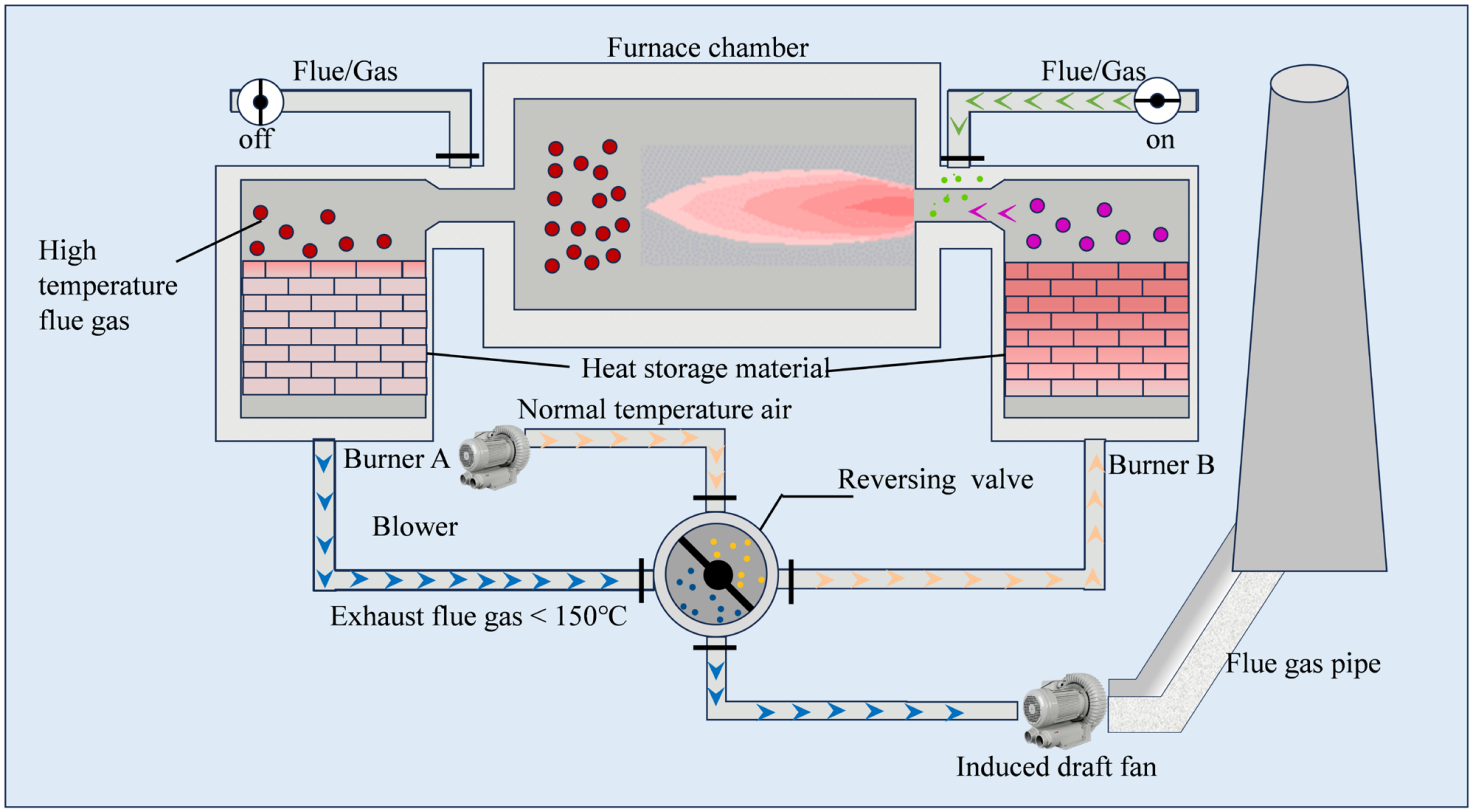
The structure and operational mechanism of regenerative aluminum smelting furnace.

The regenerative aluminum melting furnace serves as an energy-efficient system designed for smelting aluminum and its alloys. This equipment utilizes a regenerative combustion mechanism that captures waste heat from high-temperature furnace flue gases to preheat incoming combustion air, thereby enhancing thermal efficiency while minimizing energy consumption. As illustrated in Fig. 1, the system’s architecture comprises five key components: regenerative burners with integrated ceramic heat accumulators, furnace chamber, flow-direction control valves, air/flue gas pipe, and exhaust system^28,29^.

Figure 1 illustrates the paired operational configuration of Burners A and B in the regenerative furnace system. During operation, ambient-temperature combustion air is directed through the directional control valve into Burner B’s ceramic regenerator, where it undergoes preheating to near-furnace temperatures (800-1,100°C). This heated air subsequently enters the combustion chamber, entraining furnace flue gases to form oxygen-depleted combustion environments ($<21\%\ O_2$ concentration). Fuel injection initiates combustion under these oxygen-lean conditions while simultaneously enabling flue gas redirection through Burner A. The high-temperature exhaust gases (1,200-1,400°C) transfer thermal energy to Burner A’s ceramic regenerator before being discharged below 150°C. A periodic switching mechanism activates when regenerators reach thermal saturation (typically every 5–8 minutes), alternating operational roles between burners to maintain continuous heat recovery efficiency.

**Table 1:** Input variables of the aluminum smelting model.

No.	Input variable	Description
1	M-Temp	Material Temperature
2	Furnace-P	Furnace Pressure
3	C12-AF	Combustion Air Flow of No.12
4	C12-PD	Combustion Air Pressure Difference of No.12
5	C34-AF	Combustion Air Flow of No.34
6	C34-T	Combustion Air Temperature of No.34
7	C34-PD	Combustion Air Pressure Difference of No.34
8	FG34-PD	Fuel Gas Pressure Difference of No.34
9	GAF34	Gas Air-Fuel Ratio of No.34
10	B1-ET	Exhaust Gas Temperature of B1
11	B2-ET	Exhaust Gas Temperature of B2
12	B3-ET	Exhaust Gas Temperature of B3
13	B4-ET	Exhaust Gas Temperature of B4

**Table 2:** Sensor types and parameters.

Sensor type	Flow meter	Differential pressure gauge	Valve position indicator	Thermocouple
Range	0–15m³/h	0–10,000Pa	0–100%	0–1200°C
Accuracy	±1%	±0.5%	±1%	±0.5%
Response Time	0.2s	0.1s	0.1s	0.5s

**Fig. 2:**
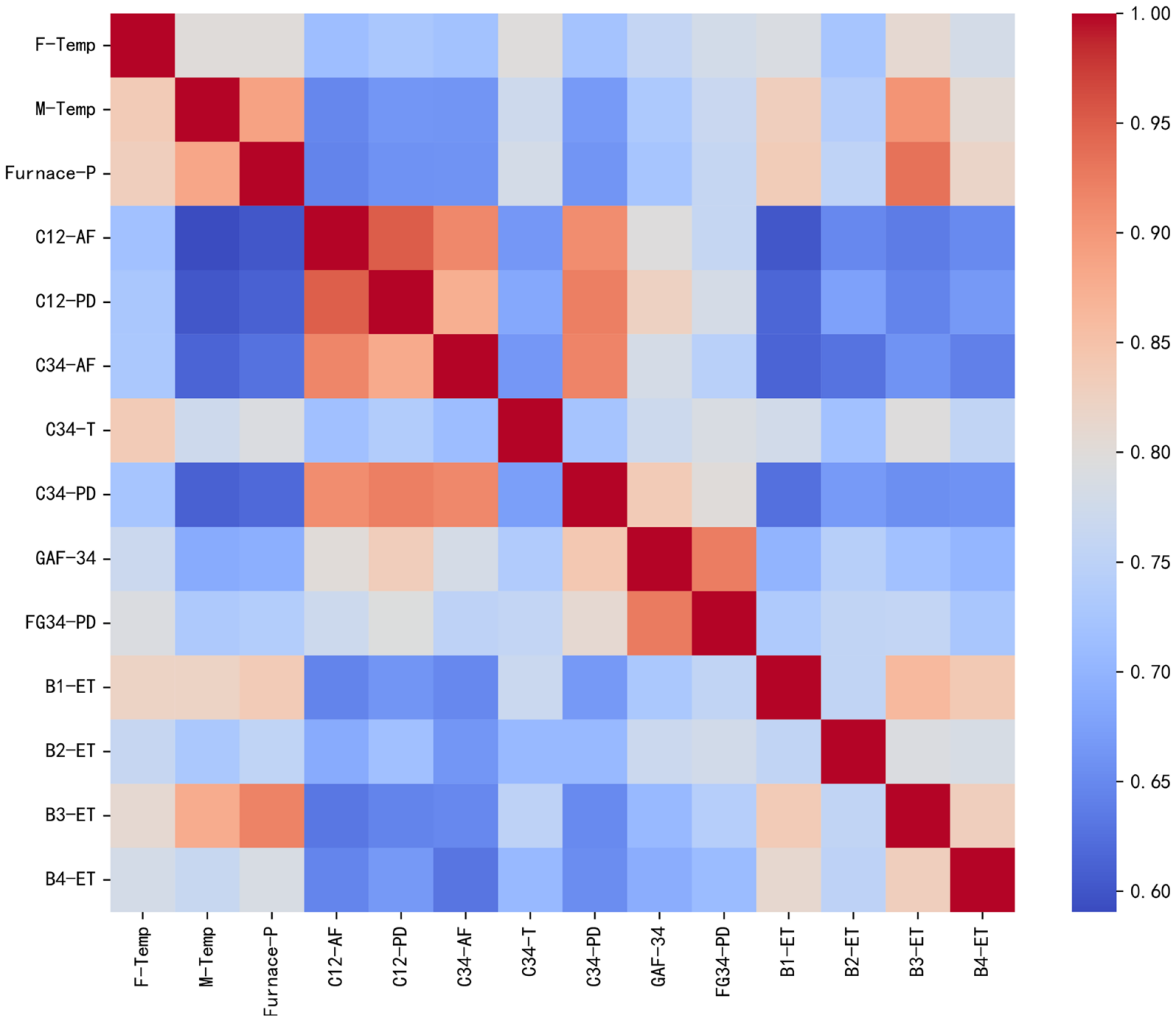
The correlations among variables through grey relational analysis.

Furnace temperature constitutes a key state variable in aluminium smelting, directly determining production cycle time and final product quality. This thermal quantity exhibits coupled dynamics with four operational inputs—gas-flow rate, combustion-air flow rate, combustion-air pressure differential and exhaust-gas temperature—listed in Table 1 and acquired by the sensor suite in Table 2. The correlations among them are obtained through grey relational analysis of the variables, as illustrated in Fig. 2.

Among them, the gas-to-air volumetric ratio dominates combustion efficiency: excess air creates an oxygen-rich flame, increases convective losses and lowers furnace temperature, whereas excess fuel leads to incomplete combustion and higher operating cost. During exhaust, a ceramic-ball regenerator recovers heat from the flue gas before directional-valve discharge; consequently, exhaust-gas temperature becomes an auxiliary control variable whose tight regulation is essential for minimising energy dissipation. Co-ordinated manipulation of combustion parameters and regenerative heat-exchange dynamics is therefore required to maintain thermal stability and maximise energy efficiency throughout the smelting campaign.

## Methodology

**Fig. 3:**
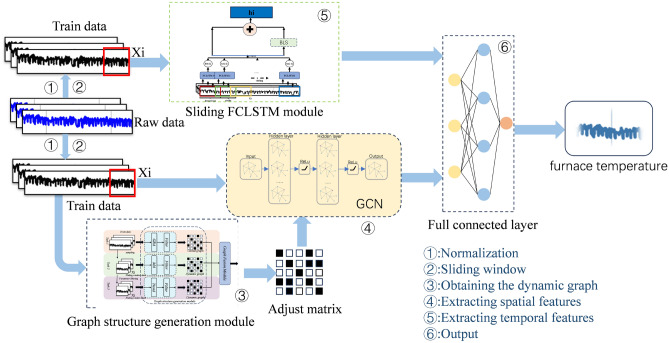
The structure of the whole model.

In this section, we present our proposed DYGCN-SLSTM soft sensor framework. The pipeline of our proposed model is shown in Fig. 3. The proposed model architecture consists of two core modules: temporal feature extraction, which captures dynamic evolution along the time axis, and spatial feature extraction, which aggregates sensor-to-sensor relationships encoded by an adaptive graph. First, a sliding window generates a single sample that is simultaneously fed into two parallel branches. In the temporal branch, a sliding LSTM extracts window-level hidden states. In the spatial branch, the graph-generation module computes the adaptive adjacency matrix by jointly assessing inter-sequence distances, pairwise trend-sequence similarities, and the global connectivity pattern. The resulting node features and adjacency matrix are then forwarded to a GCN to produce spatial embeddings. Finally, the concatenated temporal and spatial representations are passed through a fully-connected layer to yield the real-time prediction.

The detailed procedures of the DYGCN-SLSTM are as follows:

Step1: Standardize the data with maximum-minimum normalization approach. The definition of maximum-minimum normalization is as follows:

1 $$\begin{aligned} x_{\text {norm}}=\frac{x-\min (x)}{\max (x)-\min (x)} \end{aligned}$$

Denote the normalized data as $X = \{ [{x_{1h}},{x_{2h}},...,{x_{nh}}]\} _{h = 1}^H$, where *H* is the number of sampling points and *n* is the input variables count.

Step2: Apply a sliding window (size *l* and stride $s = 1$) to *X* to obtain the $w-th$ input data $X_w^L = \{ [{x_{1h}},{x_{2h}},...,{x_{nh}}]\} _{h = ws}^{ws + l - 1}$.

Step3: Use the Graph structure fusion module to generate the dynamic adjacency matrix, obtaining the dynamic graph ${G_w} = (V,E,A,X_w^L)$.

Step4: The GCN extracts spatial features $Z_w$ from the dynamic graph ${G_w}$.

Step5: The sliding LSTM extracts the temporal features $T_w$. The process of the sliding LSTM is as follows:

2 $$\begin{aligned} {h_n} = LSTMn(X_w^L[ns:ns + k]) \end{aligned}$$

3 $$\begin{aligned} {T_{_w}} = fc(concat[{h_0}[ - 1],......,{h_n}[ - 1]]) \end{aligned}$$

Where ${h_0}$- ${h_n}$ are the hidden state of each LSTM cell. $LST{M_\mathrm{{i}}}$ means the LSTM cells that match the sliding window. $X_w^L[ns:ns + k]$ means the nth set of sliding window sampled from $X_i$, and $X_i$ is sliding window data sampled from normalized data. ${h_n}[ - 1]$ is the last slice of hidden state ${h_n}$. *fc* means fully connected layer. ${T_{_w}}$ is the final input of $X_i$.

Step6: The prediction result ${\widehat{y}_t}$ is eventually outputted after passing through a fully connected layer. ${\widehat{y}_t} = \sigma (W({Z_w} + {T_w}) + b)$.

### A. Dynamic graph convolutional network (DYGCN)

Graph Convolutional Networks (GCNs) are specialized neural networks designed to handle complex graph-structured data. In contrast to traditional Convolutional Neural Networks (CNNs), which are optimized for processing data in regular, grid-like formats such as images, GCNs are uniquely constructed to manage the non-Euclidean and often irregular structure of graph data. Within this graphical framework, nodes represent distinct entities, while edges represent the relations among them. GCNs achieve effective learning over graphs by propagating, transforming, and aggregating features from nodes and their neighbors across the graph’s topology, thereby enabling the effective extraction of patterns and insights embedded within the complex relational structures of graph data.

Graph Convolutional Networks (GCNs) generate node embeddings by simultaneously leveraging graph topology and node attributes. Let $G=(V,E)$ be an *n*-node graph with adjacency matrix $\textbf{A}\in \mathbb {R}^{n\times n}$ and node feature matrix $\textbf{H}^{(l)}\in \mathbb {R}^{n\times d_{l}}$ at layer *l*. To ensure self-loops are included, we define $\tilde{\textbf{A}}=\textbf{A}+\textbf{I}_{n}$ and construct the diagonal degree matrix $\tilde{\textbf{D}}\in \mathbb {R}^{n\times n}$ with entries $\tilde{D}_{ii}=\sum _{j}\tilde{A}_{ij}$. The normalized aggregation operator

4 $$\begin{aligned} \hat{\textbf{A}} = \tilde{\textbf{D}}^{-1/2}\tilde{\textbf{A}}\tilde{\textbf{D}}^{-1/2} \end{aligned}$$

prevents feature scale explosion via symmetric normalization. The $(l+1)$-th layer output is then

5 $$\begin{aligned} \textbf{H}^{(l+1)} = \sigma \!\left( \hat{\textbf{A}}\textbf{H}^{(l)}\textbf{W}^{(l)}\right) \end{aligned}$$

Where $\textbf{W}^{(l)}\in \mathbb {R}^{d_{l}\times d_{l+1}}$ is a trainable weight matrix and $\sigma (\cdot )$ denotes a nonlinear activation function (e.g., ReLU or sigmoid). Stacking *L* such layers enables the model to capture *L*-hop relational information for downstream tasks.

**Fig. 4:**
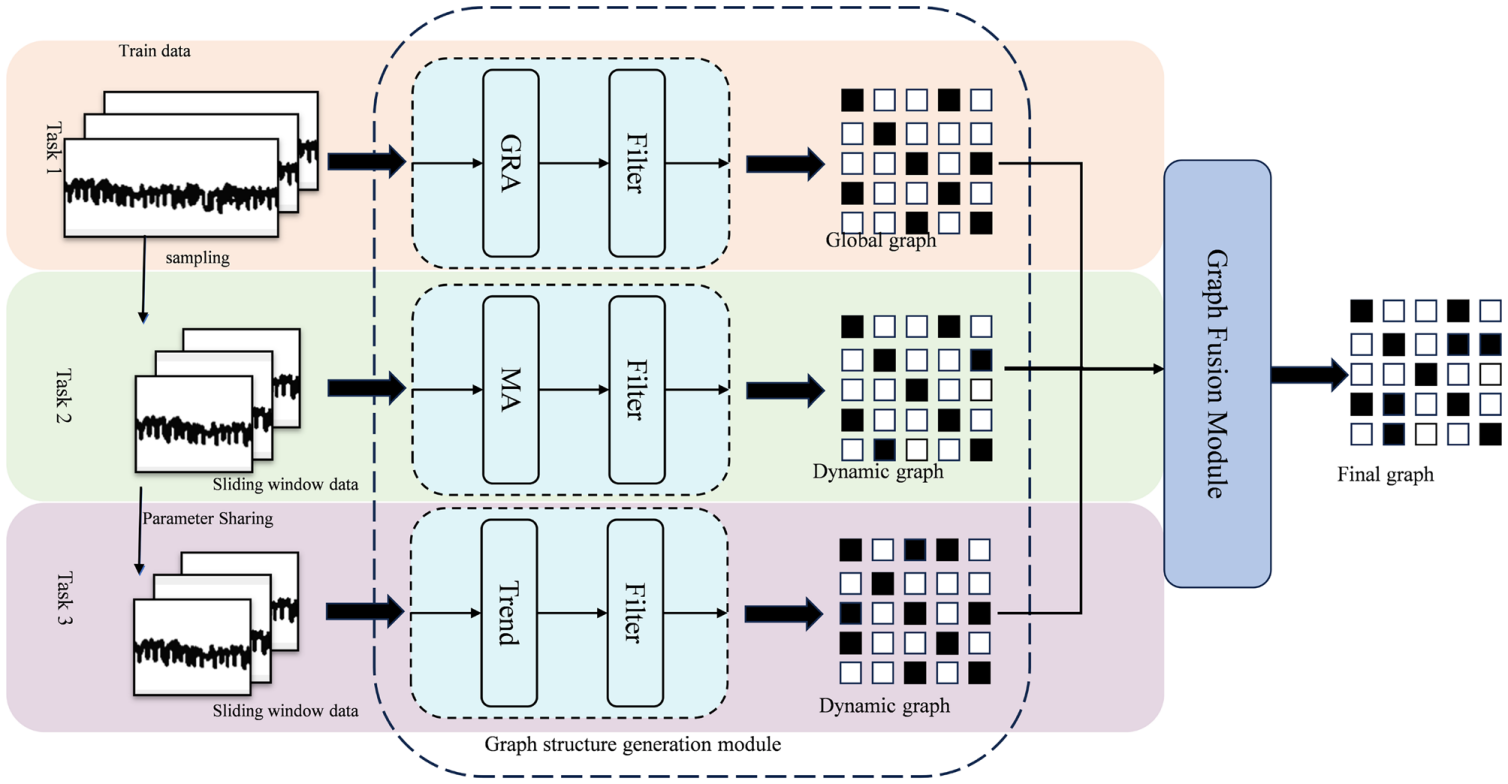
The structure of the graph structure generation module.

In practice, inter-node connectivity evolves over time, whereas standard GCNs assume a time-invariant adjacency matrix. This limitation prevents the model from attenuating task-irrelevant signals, degrading the effectiveness of representation learning. To address this issue, a dynamic-graph GCN that (i) infers time-varying adjacency and (ii) integrates the updated graph for spatial-feature extraction is proposed. The adaptive connectivity inference and integration pipeline are illustrated in Fig. 4.

Figure 4 summarizes the gray relational analysis (GRA) performed on the training set to construct the global adjacency matrix A0 for Task 1. A0 serves as a prior that restricts the feasible space of node-wise adjacency values, thereby suppressing spurious edges at arbitrary time steps. The GRA-based computation of A0 over the dataset is given by:

6 $$\begin{aligned} A0_{ij/ji} = GRA({X_i},{X_j}) \end{aligned}$$

7 $$\begin{aligned} \{\begin{array}{l} \operatorname {coeff}_{i j}(t)=\frac{\min _{i j}+\alpha \cdot \max _{i j}}{\left| x_{t}^{i}-x_{t}^{j}\right| +\alpha \cdot \max _{i j}}, t=1,2, \cdots , T . \\ \operatorname {G}RA({X_i},{X_j}) =\frac{1}{T} \sum _{t=1}^{T} \operatorname {coeff}_{i j}(t) \end{array} \end{aligned}$$

8 $$\begin{aligned} A0 = \left\{ \begin{array}{l} A_{ij} = 1,{\hspace{1.0pt}} {\hspace{1.0pt}} {\hspace{1.0pt}} {\hspace{1.0pt}} {\hspace{1.0pt}} {\hspace{1.0pt}} if{\hspace{1.0pt}} {\hspace{1.0pt}} {\hspace{1.0pt}} {\hspace{1.0pt}} A0_{ij} \ge 0.75\\ A_{ij} = 0,{\hspace{1.0pt}} {\hspace{1.0pt}} {\hspace{1.0pt}} {\hspace{1.0pt}} if{\hspace{1.0pt}} {\hspace{1.0pt}} {\hspace{1.0pt}} {\hspace{1.0pt}} A0_{ij} < 0.75 \end{array} \right. \end{aligned}$$

Where $\min _{i j}$ means the minimum of all elements in $\left| x_{t}^{i}-x_{t}^{j}\right|$, $\max _{i j}$ means the maximum of all elements in $\left| x_{t}^{i}-x_{t}^{j}\right|$. $\operatorname {coeff}_{i j}(t)$ is the correlation coefficient of ${X_i}$ and ${X_j}$ at *t* time; $\alpha$ is the resolution factor and is set as 0.5. *T* is the length of time series.

Next, Task 2 computes the Mahalanobis distance between the current feature vectors to construct the dynamic adjacency matrix A1 in real time. This scale-invariant metric captures pairwise temporal dependencies among features without being influenced by their magnitudes. The computation of A1 over the dataset is given by:

9 $$\begin{aligned} Ma({X_i},{X_j})=\sqrt{\left( \textbf{X}_{i}-\textbf{X}_{j}\right) ^{\top } \Sigma ^{-1}\left( \textbf{X}_{i}-\textbf{X}_{j}\right) } \end{aligned}$$

10 $$\begin{aligned} \Sigma =\frac{1}{T-1} \sum _{t=1}^{T}\left( \textbf{X}_{t}-\overline{\textbf{X}}\right) \left( \textbf{X}_{t}-\overline{\textbf{X}}\right) ^{\top } \end{aligned}$$

11 $$\begin{aligned} \overline{\textbf{X}}=\frac{1}{T} \sum _{t=1}^{T} \textbf{X}_{t} \end{aligned}$$

12 $$\begin{aligned} A1_{ij/ji}=1-\frac{ Ma({X_i},{X_j})-\min ( Ma({X_i},{X_j}))}{\max ( Ma({X_i},{X_j}))-\min ( Ma({X_i},{X_j}))} \end{aligned}$$

13 $$\begin{aligned} A1_{ij/ji}= \left\{ \begin{array}{l} 1,{\hspace{1.0pt}} {\hspace{1.0pt}} {\hspace{1.0pt}} {\hspace{1.0pt}} {\hspace{1.0pt}} if{\hspace{1.0pt}} {\hspace{1.0pt}} {\hspace{1.0pt}} {\hspace{1.0pt}} {\hspace{1.0pt}} {\hspace{1.0pt}} A1_{ij/ji} \ge 0.8\\ 0,{\hspace{1.0pt}} {\hspace{1.0pt}} {\hspace{1.0pt}} {\hspace{1.0pt}} if{\hspace{1.0pt}} {\hspace{1.0pt}} {\hspace{1.0pt}} {\hspace{1.0pt}} A1_{ij/ji} < 0.8 \end{array} \right. \end{aligned}$$

Where $\Sigma$ is covariance matrix estimated from the set of all time-series. $\Sigma ^{-1}$ denotes inverse of the covariance matrix. $\overline{\textbf{X}}$ is the mean vector across all time-series. $^{\top }$ means transpose. Formula 12 is to convert the smaller the better distance measure into a larger the better similarity fraction

**Fig. 5:**
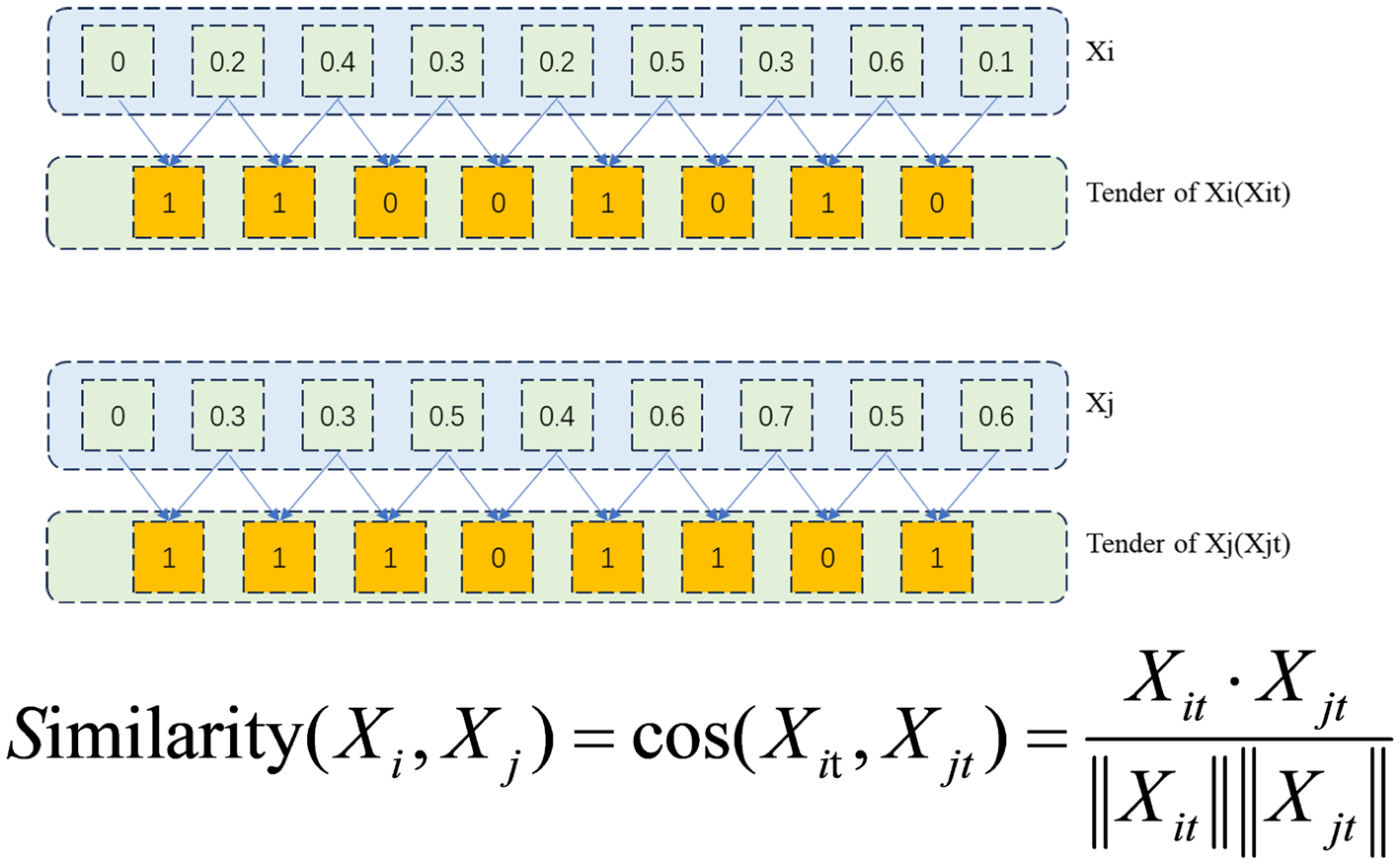
The detail method of producing A2.

Unlike the Mahalanobis distance, Task 3 computes the binary trend sequence similarity between feature vectors to construct the dynamic adjacency matrix A2 in real time. The detailed procedure is illustrated in Fig. 5. For each feature, we generate a binary sequence by comparing its value at the current time step with that at the previous step; if the value remains unchanged or increases, the bit is set to 1, otherwise to 0. The similarity between two sequences is then defined as their cosine similarity. If a sequence is all zeros, its similarity to any other sequence is defined as 0. The computation of A2 over the dataset is formulated below:

14 $$\begin{aligned} A2_{ij} = \mathrm{{similarity(}}{\mathrm{{X}}_\mathrm{{i}}}\mathrm{{,}}{\mathrm{{X}}_\mathrm{{j}}}\mathrm{{)}} \end{aligned}$$

15 $$\begin{aligned} A2_{ij} = \left\{ \begin{array}{l} 1,ifA2_{ij} \ge 0.5\\ 0,ifA2_{ij}< 0.5 \end{array} \right. \end{aligned}$$

The final graph (A) is derived from the real-time aggregation of all As. The computation of the entire set of As for data set can be expressed as follows:

16 $$\begin{aligned} A = \mathrm{{attention}}(A0,A1,A2) \end{aligned}$$

**Fig. 6:**
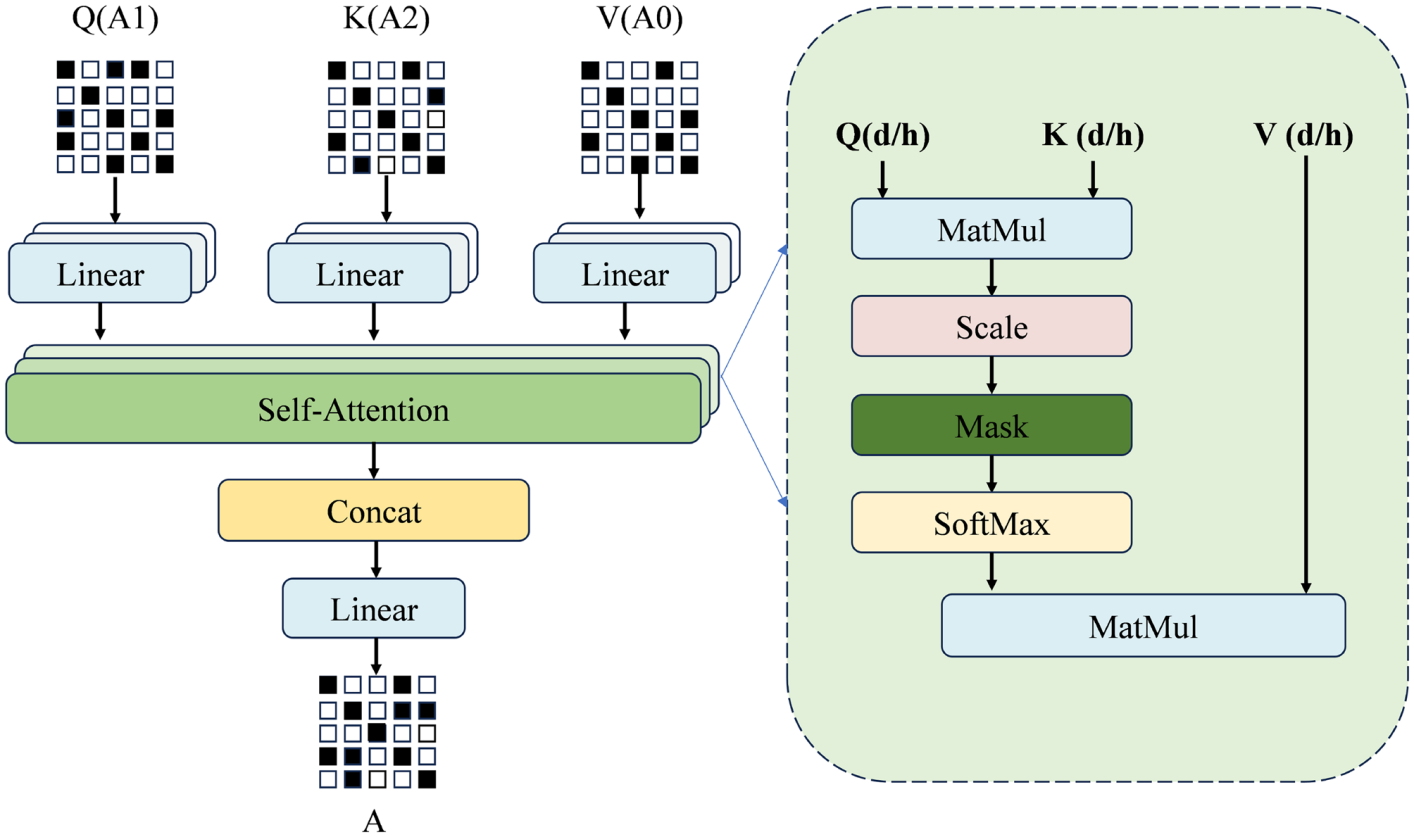
The detail method of graph fusion.

The final adjust matrix is learned by attention mechanism with three matrix above in real time. Figure 6 shows the detail method of graph fusion, where *d* represents the dimensions of $A_s$, and *h* denotes the total number of attention heads. The detail method of graph fusion can be expressed as follows:

17 $$\begin{aligned} \textrm{Attention}(Q,K,V)=\textrm{softmax}\left( \frac{Q\cdot K^\textrm{T}}{\sqrt{d_k}}\right) \cdot V \end{aligned}$$

18 $$\begin{aligned} \textrm{Head}_i=\textrm{Attention}\left( Q\cdot W_i^Q,K\cdot W_i^K,V\cdot W_i^V\right) \end{aligned}$$

19 $$\begin{aligned} \textrm{MultiHead}(X)=\textrm{Concat}(\textrm{head}_1,\ldots ,\textrm{head}_H)W_O \end{aligned}$$

Where $Attention(\cdot )$ is the function for self-attention calculation; *Q*, *K*, and *V* are the query, key, and value matrices, respectively; $^\top$ denotes the matrix transpose operation; $d_k$ denotes the dimension of *K*; $\text {softmax}(\cdot )$ is the normalization function used to convert scores into probabilities. $Head_i$ denotes the output of the *i*-th attention head, *h* denotes the total number of attention heads, and $W_i^Q$, $W_i^K$, $W_i^V$ are matrices used to project *Q*, *K*, and *V* into a higher-dimensional representation.$\textrm{MultiHead}(X)$ is the function for multi-head self-attention calculation, $W^O$ is the output projection matrix.

In figure fig:attention, global graph (A0) is used as Value (V) input in the graph fusion mechanism to ensure that the model can make full use of the key features of the whole data set. These characteristics are critical to capturing the overall structure and patterns of the data. The stability and richness of global information provide a stable reference point for the model, which is helpful to improve the robustness and prediction accuracy of the model.

A two-layer GCN will capture the spatial features of data, and the features will be processed in next model.

### B. Sliding FCLSTM

LSTM is a variant of recurrent neural networks (RNNs) specifically designed to mitigate gradient vanishing or explosion. To capture short-term temporal dependencies more effectively, we adopt a fully-connected LSTM that additionally employs a gating mechanism to filter features. The three gates of the LSTM are defined as follows:

20 $$\begin{aligned} f_t=\sigma (W_f\cdot [c_{t-1},h_{t-1},A_t]+b_f) \end{aligned}$$

21 $$\begin{aligned} i_t=\sigma (W_i\cdot [c_{t-1},h_{t-1},A_t]+b_i) \end{aligned}$$

22 $$\begin{aligned} o_t=\sigma (W_o\cdot [c_{t},h_{t-1},A_t]+b_o) \end{aligned}$$

where, $W_f$,$W_i$,$W_o$ are the weight matrices of LSTM’s gates, with $b_f$, $b_i$, $b_o$ as their corresponding biases. $[c_{t-1},h_{t-1},A_t]$ means that three vectors are joined into a longer vector. $\sigma$ represents the Sigmoid function.

**Fig. 7:**
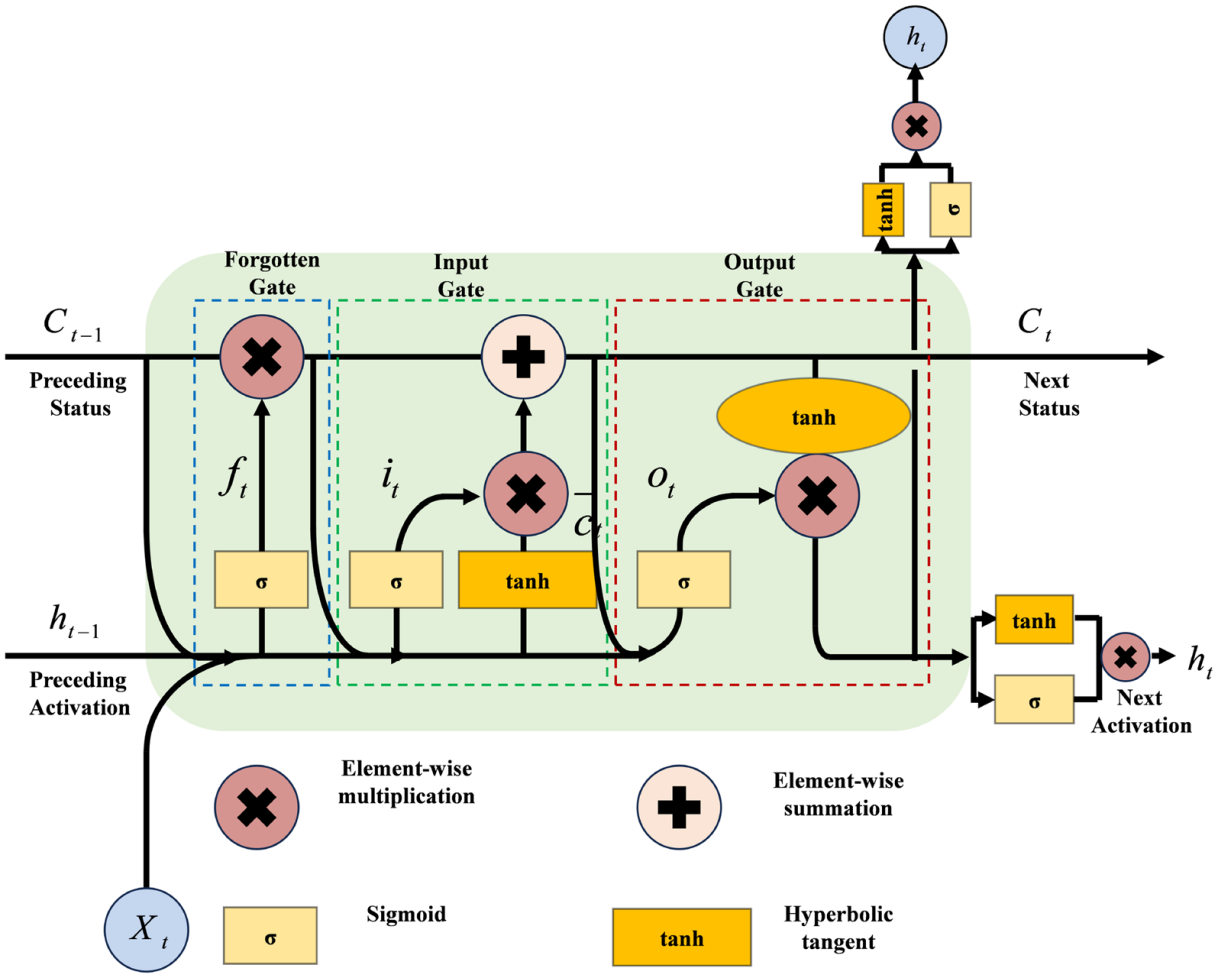
Diagram of fully-connected LSTM network.

The LSTM network structure is shown in Fig. 7, which is computed as follows:

The three gates (forget gate, input gate, and output gate) are computed based on the external state $h_{t-1}$ from the previous time step, the candidate state $\overline{c}_t$, and the input $X_t$ at the current time step.

The memory cell $c_t$ is updated by the The forgetting gate $f_t$ and the input gate $i_t$ working together.

The output gate $o_t$ integrates internal state information and transfers it to the external state $h_t$.

**Fig. 8:**
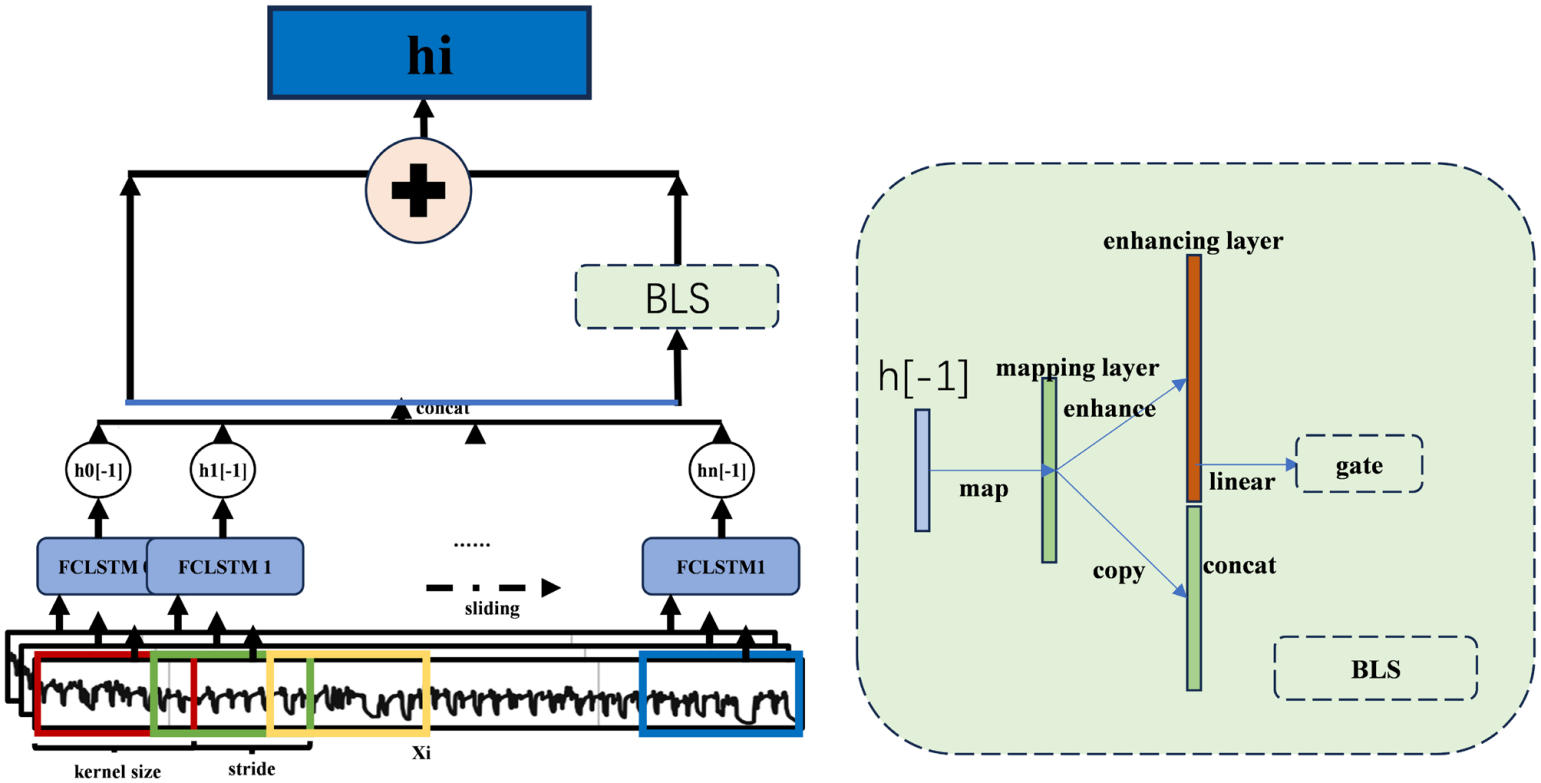
The structure of the SLSTM.

LSTM can predict the state of the next moment relatively accurately when dealing with time series with stable changes. However, most engineering datasets contain variables with extremely large numerical variations, making it difficult for LSTM to represent these changes in a timely manner and thus failing to meet industrial requirements. SLSTMs have a sliding network; Fig. 8 shows the framework of sliding fully-connected LSTM. The sliding LSTM extracts the temporal features $T_w$. The process of the sliding LSTM is as follows:

23 $$\begin{aligned} {h_0} = LST{M_0}(X_w^L[0:0 + k]) \end{aligned}$$

24 $$\begin{aligned} {h_n} = LSTMn(X_w^L[ns:ns + k]) \end{aligned}$$

25 $$\begin{aligned} {T_{_w}} = fc(concat[{h_0}[ - 1],......,{h_n}[ - 1]]) \end{aligned}$$

26 $$\begin{aligned} B L S\left( T_{w}\right) =\left\{ \begin{array}{l} Z=\sigma \left( T_{w} \cdot W_{z}\right) \\ H=\sigma \left( Z \cdot W_{h}\right) \\ \text{ out } =\sigma [Z, H] \end{array}\right. \end{aligned}$$

27 $$\begin{aligned} {h_{_i}} = BLS(T_{_w})*W + T_{_w} \end{aligned}$$

Where ${h_0}$- ${h_n}$ are the hidden state of each LSTM cell. $LST{M_\mathrm{{i}}}$ means the LSTM cells that match the sliding window. $X_w^L[ns:ns + k]$ means the nth set of sliding window sampled from $X_i$, and $X_i$ is sliding window data sampled from normalized data. ${h_0}[ - 1]$ is the last slice of hidden state ${h_0}$. *fc* means fully connected layer. ${T_{_w}}$ is the final input of $X_i$. $W_z$ and $W_h$ are weight matrix of mapping layer and enhancing layer in the BLS block, which are randomly initialized and frozen, *W* is the weight matrix.

BLS block is inspired by broad learning system. But it’s not the traditional expanded hidden unit: it’s the random mapping and nonlinear enhancement of hidden features. The block constructs a high-dimensional, information-rich embedding, which reflects the internal structure of the data. A learnable gating mechanism controls effective signal propagation forward. This block not only deepens the model’s perception of subtle patterns, but also gives the model an adaptive attention to the feature correlation.

In Fig. 8, $\textbf{X}_i\in \mathbb {R}^{w\times d}$ denotes the *i*-th sliding-window sub-sequence of length *w* sampled from the training set. Unlike CNNs, the proposed model employs a bank of *K* kernels, each dedicated to the sub-sequence covered by its receptive field. With stride *s*, the kernel bank produces $M=\lfloor (L-w)/s \rfloor +1$ hidden vectors $\{\textbf{h}_k\}_{k=1}^{K}$, which are concatenated into $\textbf{H}\in \mathbb {R}^{KM}$. After feeding $\textbf{H}$ into the BLS block, a residual connection is added between the BLS output and the original LSTM hidden state to yield the final prediction. Experimental results demonstrate that the proposed architecture significantly improves short-term feature detection compared with vanilla LSTM. By re-weighting per-time-step LSTM outputs through learned attention scores, the model simultaneously captures long-range dependencies and localized transient events within the sequence.

## Experiments

Validation experiments were conducted using industrial operational data from a regenerative aluminum smelting furnace for summer and winter, divided into two datasets by season (dataset_summer, D-S, and dataset_winter, D-W), with furnace temperature as the predictive target. These two datasets contains temporal records acquired at 5-minute sampling intervals, comprising 3,000 data samples. These two 3,000 points are a randomly selected subset from two complete datasets of approximately 20,000 points, with the original intention of testing the model’s generalization ability under limited data. Through grey relational analysis, 14 process parameters demonstrating strongest system correlation were identified, with furnace temperature designated as the target parameter and the remaining 13 variables assigned as input features. To ensure methodological rigor, these datasets were partitioned into stratified subsets: 60% for model training, 20% for hyperparameter validation, and 20% for final performance evaluation, enabling comprehensive assessment under varying operational conditions.

To further assess the model’s generalization ability, an external validation was conducted using an additional dataset collected during autumn (Dataset D-A), consisting of 1,000 samples with the same 5-min sampling interval. This dataset represents operational conditions distinct from the summer and winter periods. The models previously trained on the summer (D-S) and winter (D-W) datasets were directly applied to the autumn data without any parameter adjustment, aiming to evaluate their predictive performance under unseen operational conditions.

In this study, the performance of the models is assessed utilizing Root Mean Square Error (RMSE), Mean Absolute Error (MAE), Mean Absolute Percentage Error (MAPE), the coefficient of determination (R²), and Maximum Absolute Error (MAX). These metrics are formalized as follows:

28 $$\begin{aligned} RMSE = {\hspace{1.0pt}} {\hspace{1.0pt}} {\hspace{1.0pt}} \sqrt{{\hspace{1.0pt}} {\hspace{1.0pt}} {\hspace{1.0pt}} \frac{1}{n}{{\sum \limits _{i = 1}^n {({y_i} - {{\widehat{y}}_i})} }^2}} \end{aligned}$$

29 $$\begin{aligned} MAE = {\hspace{1.0pt}} {\hspace{1.0pt}} {\hspace{1.0pt}} \frac{1}{n}\sum \limits _{i = 1}^n {\left| {{y_i} - {{\widehat{y}}_i}} \right| } \end{aligned}$$

30 $$\begin{aligned} MAPE = {\hspace{1.0pt}} {\hspace{1.0pt}} {\hspace{1.0pt}} \frac{1}{n}\sum \limits _{i = 1}^n {\left| {\frac{{{y_i} - {{\widehat{y}}_i}}}{{{y_i}}}} \right| } \end{aligned}$$

31 $$\begin{aligned} R2 = 1 - \frac{{\frac{1}{n}{{\sum \limits _{i = 1}^n {({y_i} - {{\widehat{y}}_i})} }^2}}}{{\frac{1}{n}{{\sum \limits _{i = 1}^n {({y_i} - {{\overline{y} }_i})} }^2}}} \end{aligned}$$

32 $$\begin{aligned} \text {MAX} = \max _{i=1}^{n} |y_i - \hat{y}_i| \end{aligned}$$

Where ${y_i}$ represents the observed value, ${\widehat{y}_i}$ represents the predicted value, ${\overline{y} _i}$ is the means of observed values and *n* is the number of observations.

In the parameter tuning process of the DYGCN‑SLSTM algorithm, we first determined the time window size L and the sliding window size W. For the time window size, based on the operating cycle of the aluminum smelting furnace and empirical knowledge, it was preliminarily set to 60 minutes, corresponding to a window length of 12 sampling points. For the sliding window size, based on the feedback time after changes in furnace operating conditions, it was preliminarily set to 15 minutes, corresponding to a window length of 3 sampling points. These parameters were then finalized through trial‑and‑error experiments conducted on the test dataset D-W. The performance evaluation results of the model under different L values are presented in Table 3, and the results under different W values are shown in Table 4. Analysis indicates that the model achieves optimal performance when the time window L is set to 12 and the sliding window size W is set to 4.

**Table 3:** Comparison of model performance with different L on D-W.

L	RMSE	MAPE	MAE	R2	MAX
10	3.3194	0.0045	4.7053	0.9971	14.8223
11	3.4355	0.0048	4.5879	0.9972	14.4538
12	3.0541	0.0040	4.3292	0.9985	13.4692
13	3.5229	0.0044	4.4960	0.9979	13.8942
14	3.2905	0.0045	4.6643	0.9975	15.5646
15	3.7360	0.0052	5.2957	0.9962	15.3134

**Table 4:** Comparison of model performance with different W on D-W.

W	RMSE	MAPE	MAE	R2	MAX
3	3.6194	0.0053	5.7543	0.9965	15.8223
4	3.0541	0.0040	4.3292	0.9985	13.4692
5	3.4325	0.0048	4.7549	0.9975	14.9538
6	3.9299	0.0049	4.4960	0.9966	15.8942

**Table 5:** Comparison of prediction accuracy with different models on D-S and D-W.

Dataset	Methods	RMSE	MAE	MAPE	R2	MAX
D-W	LSTM	11.9857	9.0593	0.0115	0.9905	35.4811
	TCN	11.2835	6.8182	0.0102	0.9940	55.6413
	TimeXer	9.6067	6.8945	0.0087	0.9960	30.0765
	PatchTST	7.1918	4.6564	0.0060	0.9977	34.4592
	DYGCN-SLSTM	4.3127	3.0215	0.0041	0.9985	13.4692
D-S	LSTM	2.6451	1.8012	0.0115	0.9913	7.9966
	TCN	3.9622	3.3151	0.0043	0.9923	7.7344
	TimeXer	2.4758	1.6063	0.0021	0.9965	8.4997
	PatchTST	1.7786	1.2424	0.0016	0.9982	4.6566
	DYGCN-SLSTM	1.8496	1.6111	0.0021	0.9983	4.3824

To rigorously validate the model’s efficacy, a comparative analysis was conducted against established temporal forecasting architectures (LSTM and TimeXer) alongside the proposed DYGCN-SLSTM framework. Both architectures, specifically architected for temporal sequence prediction, implement distinct forecasting paradigms: multi-horizon prediction iteratively generates sequential outputs for extended temporal windows, demonstrating utility in long-term trend projection while being susceptible to error accumulation mechanisms; whereas single-step prediction focuses on immediate next-step estimation, offering enhanced responsiveness to real-time system dynamics particularly suited for time-sensitive operational scenarios.

**Fig. 9:**
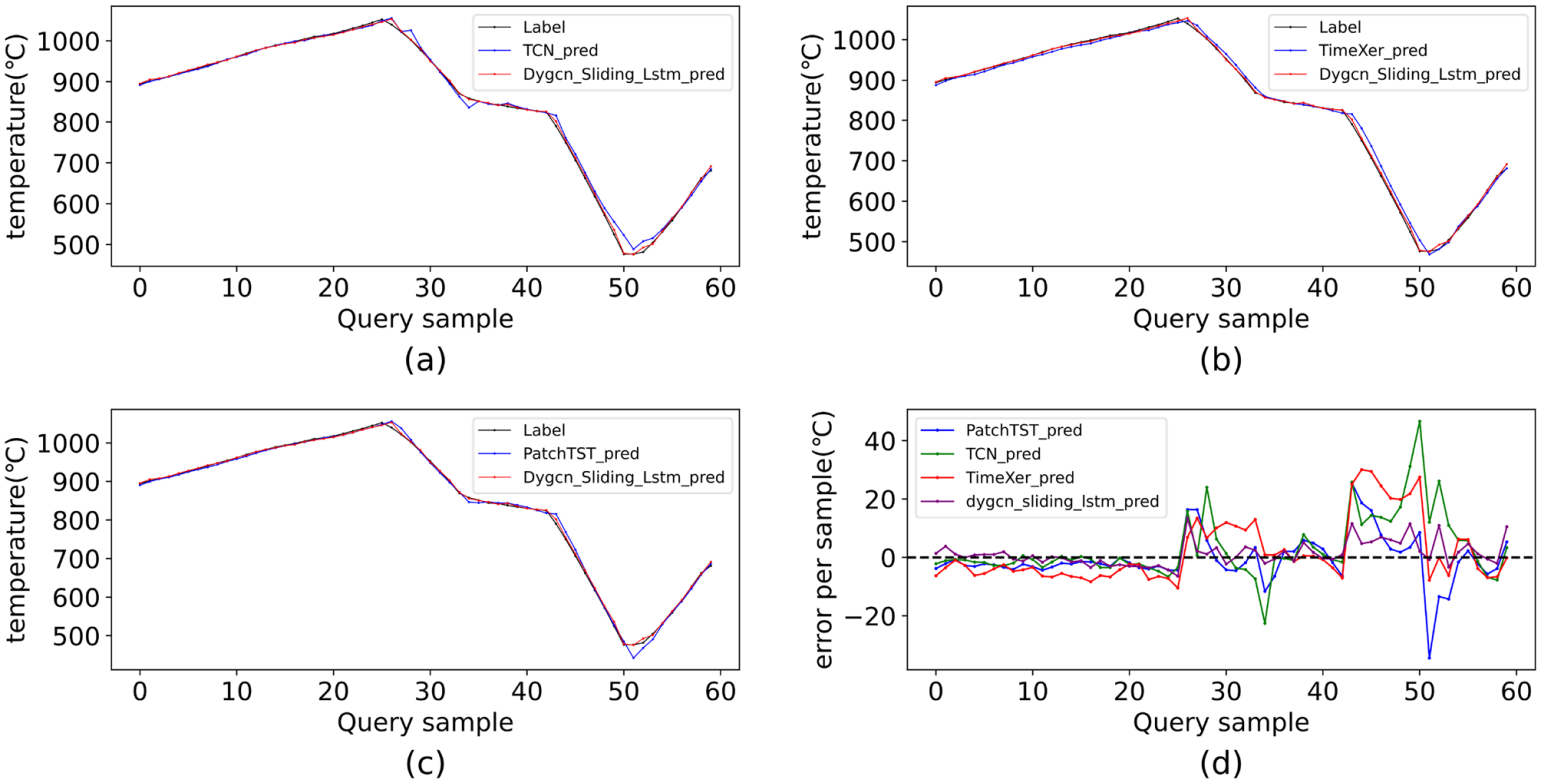
Detailed prediction results from models on D-W. (**a**) TCN; (**b**) TimeXer; (**c**) PatchTST; (**d**) error per sample.

**Fig. 10:**
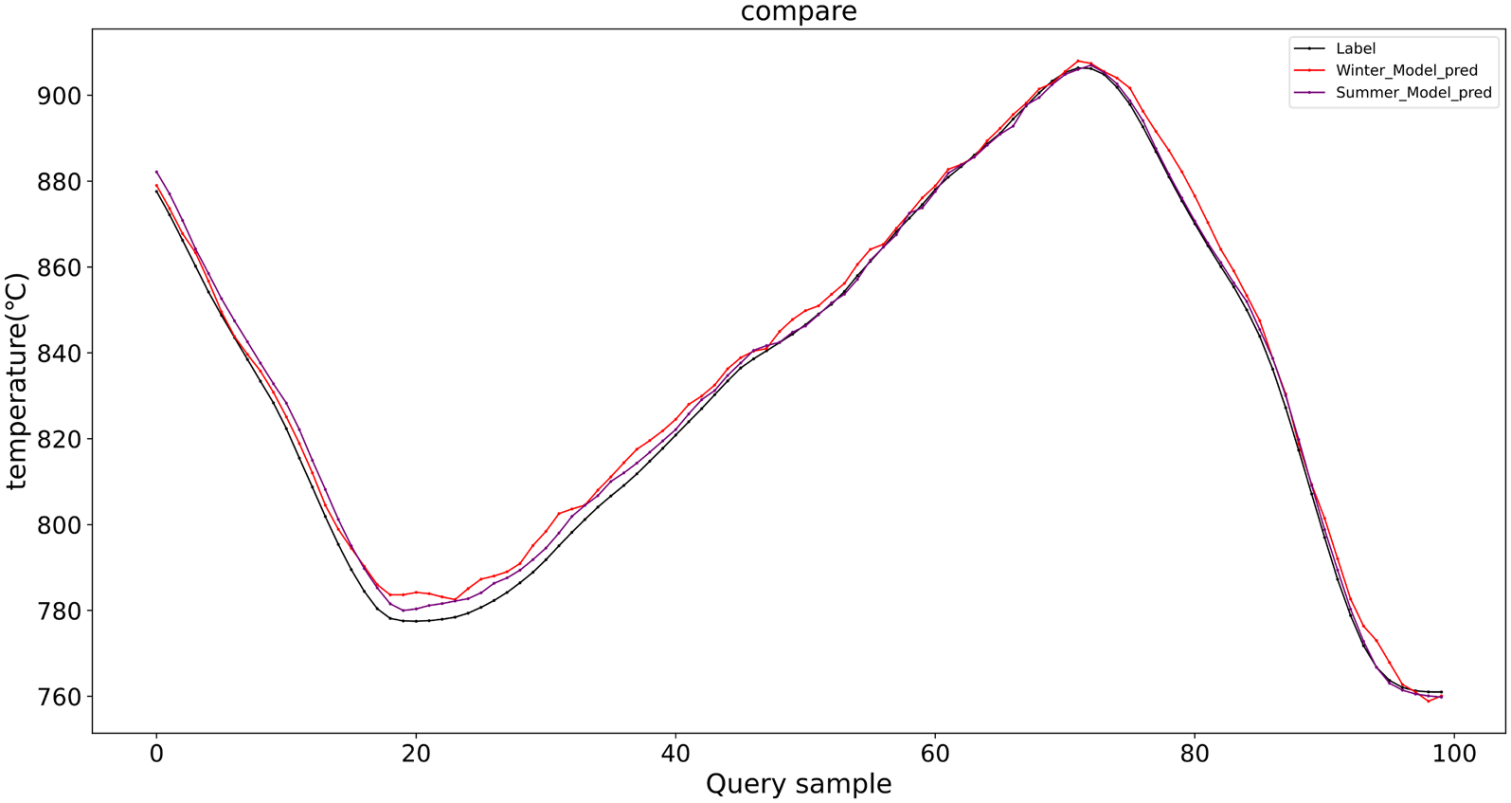
The prediction results of external validation on the autumn dataset.

In the context of time series models, the optimal parameters were empirically determined. The training epoch for all models is 200, the batchsize is 16, and the learning rate is 0.001, 0.001, 0.001, and 0.001, respectively. The final results on two datasets are presented in Table 5. A detailed comparison of prediction results between our method and each model on the testing set D-w is shown in Fig. 9. Additionally, the external validation results on the autumn dataset are summarized in Table 6, and the corresponding prediction comparisons are illustrated in Fig. 10.

**Table 6:** The external validation results on the autumn dataset.

Model	RMSE	MAE	MAPE	R2	MAX
Model trained on D-W	3.6194	3.2079	0.0053	0.9924	6.6453
Model trained on D-S	2.7532	2.1567	0.0039	0.9959	7.4896

In Table 5, the winter dataset D-W captures furnace temperature dynamics under low ambient temperature conditions. During winter, increased heat dissipation due to lower environmental temperatures necessitates a higher temperature setpoint or an elevated upper limit to maintain consistent melting efficiency. Consequently, the furnace temperature is not only generally higher but also exhibits greater fluctuation intensity and more abrupt temporal variations. The prediction performance of LSTM is relatively poor. Although LSTM can capture the correlations within time series, its ability to extract multiple features is limited. Moreover, when feature values change dramatically in a short period, LSTM struggles to reflect these changes promptly and accurately, leading to lagged prediction results. TCN achieves slightly lower RMSE and MAE than LSTM on the D-W dataset, indicating a moderate improvement in capturing temporal dependencies via dilated convolutions. However, its maximum error is substantially higher than that of other models, suggesting instability under abrupt temperature fluctuations. In contrast, TimeXer is more effective in feature extraction and has lower requirements for input features, as it can process and correct them. However, with small datasets, TimeXer cannot fully leverage its architectural strengths, and the noise in the features further weakens the model’s ability to capture temporal dependencies. PatchTST, with its unique patching mechanism and channel-independence design, has demonstrated good prediction performance in experiments. Nevertheless, the aforementioned models primarily focus on extracting temporal dependencies of variables, neglecting the dynamic changes in the connections between variables. In reality, the spatial correlations between variables also evolve over time. Quantifying this dynamic correlation in the data for training can lead to better predictions of the target sequence. DYGCN calculates the correlations in different aspects instantaneously to generate dynamic graphs of sliding window data, thereby more effectively capturing spatial features. Meanwhile, Sliding LSTM captures temporal features through parallel processing. The features computed by each LSTM unit are allocated by learnable weights to mitigate lagging. Therefore, when predicting long-span time series, DYGCN-SLSTM outperforms the other models we tested, achieving the lowest RMSE, MAE, MAPE and MAX, as well as the highest R-squared value.

On the summer dataset D-S, the prediction performance of each model presents a different pattern compared to winter. During summer, the ambient temperature is high, and the furnace body benefits from better heat retention, making it easier to maintain a high internal temperature. To reduce energy consumption and prevent overheating, the furnace temperature control target is typically set lower, and the temperature variation tends to be smoother. LSTM still suffers from limited feature extraction capability and underperforms most comparison models. TCN exhibits even higher prediction errors than LSTM, indicating poor adaptability to summer data—likely due to a mismatch between the smoother fluctuation patterns and its convolutional receptive field. TCN underperforms relative to LSTM across all metrics, implying that its fixed receptive field may not adapt well to varying operational conditions. TimeXer performs robustly, demonstrating strong feature processing capability even on small summer datasets. Notably, PatchTST achieves overall performance in summer, benefiting from its patching mechanism and channel-independence design, which are well-suited to periods of relatively stable variable relationships. DYGCN-SLSTM shows slightly higher average errors than PatchTST, but still delivers excellent goodness-of-fit and effectively suppresses extreme prediction deviations, thanks to its ability to capture real-time spatiotemporal correlations via dynamic graph convolution and parallel temporal processing with Sliding LSTM. Additionally, the model complexity of DYGCN-SLSTM is lower than that of PatchTST, making it more suitable for industrial applications.

Figure 9 clearly illustrates the performance comparison of various models in predicting operating conditions within an aluminum smelting furnace. As observed, the prediction curve of the DYGCN-SLSTM model aligns more closely with the ground truth values than those of other baseline models, demonstrating superior fitting accuracy. Notably, the maximum prediction errors of all models are predominantly concentrated during the switching phases of operating conditions. This phenomenon can be attributed to the fact that, in practical production scenarios, condition switching is occasionally subject to manual intervention, resulting in considerable uncertainty and randomness in both the timing and extent of the transitions. Consequently, traditional models often struggle to capture the critical features inherent in such dynamic processes. In contrast, DYGCN-SLSTM not only extracts temporal dependencies but also incorporates spatial feature modeling capabilities, enabling it to characterize the intrinsic correlations underlying operating condition changes from multiple dimensions. This significantly enhances the model’s adaptability to the stochastic variations in switching timing. Accordingly, Figure 9 reveals that DYGCN-SLSTM yields substantially lower maximum prediction errors, which not only substantiates its superior performance in complex industrial environments but also underscores its enhanced robustness when confronted with uncertainties and nonlinear disturbances.

Furthermore, external validation results on the autumn dataset (D-A), as summarized in Table 6 and illustrated in Fig. 10, demonstrate that the proposed DYGCN-SLSTM model maintains robust predictive performance across different seasonal conditions, irrespective of whether it is trained on summer or winter data. Both training variants achieve high accuracy, with R² values exceeding 0.99 and consistently low error metrics. The average prediction error of the two models is approximately 3 °C, which falls within the acceptable measurement error range for aluminum smelting processes. These findings confirm that the model effectively captures underlying process dynamics that generalize across seasonal variations, underscoring its strong generalization capability and practical suitability for year-round industrial deployment.

The time complexity and modeling effectiveness of each model on dataset are also given in Table 7 to further compare the prediction performance of the DYGCN-SLSTM, LSTM, TimeXer, and PatchTST.

**Table 7:** Time complexity for different models.

Model	Time complexity	RMSE	R2	MAX
LSTM	$O(BL(d_l)^2)$	11.9857	0.9905	35.4811
TimeXer	$O(BC(L/S)((d_l)^2+d_l))$	9.6067	0.9960	30.0765
PatchTST	$O(BC(L/S)^2d_l)$	7.1918	0.9977	34.4592
DYGCN-SLSTM	$O(B((N^2/2)L+N^2d_l + Ld_l^2))$	4.3127	0.9985	13.4692

*B* is batch size, *N* is the number of the nodes, *L* is the length of the sequence, *C* is the number of the models’ channels, *S* is the number of sample points the sliding window moves forward each time, $d_l$ is the dimension of the hidden layer of each model, respectively.

In Table 7, the number of nodes and the sequence length are close and significantly fewer than the dimension of the hidden layer. Consequently, the complexity of each model is mainly determined by the dimension of the hidden layer. With TimeXer and PatchTST possessing considerably larger hidden layer dimensions compared to other models, the time complexity of DYGCN-SLSTM is lower than that of TimeXer and PatchTST. And the same time, it has superior predictive effectiveness and meets the temperature measurement error requirements in practical production scenarios. Therefore, DYGCN-SLSTM is more appropriate for forecasting furnace temperatures than the other three models.

**Fig. 11:**
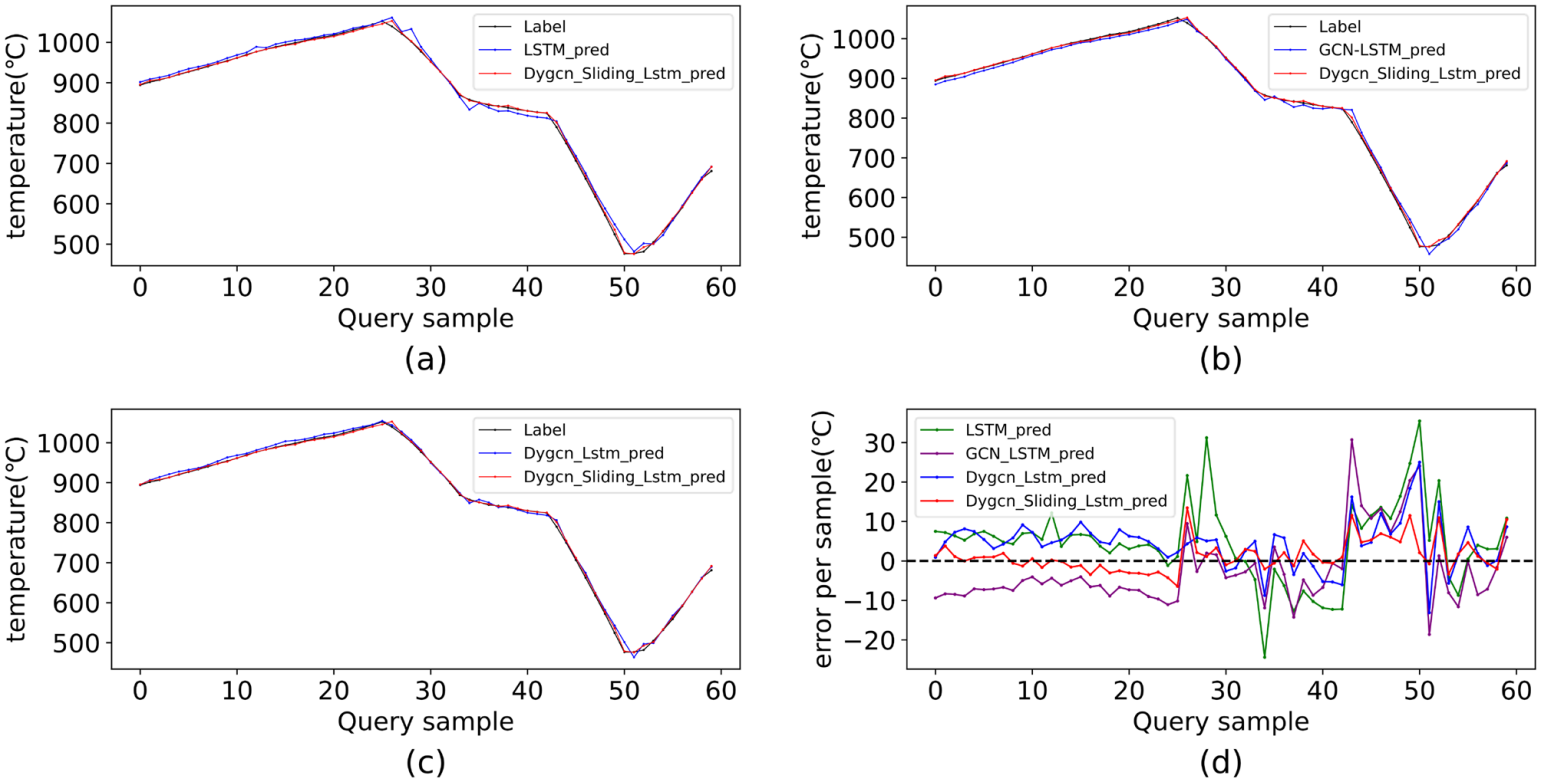
Detailed prediction results on D-W from models. (**a**) LSTM; (**b**) GCN_LSTM; (**c**) Dygcn_Sliding_Lstm; (**d**) error per sample.

To further assess the effectiveness of each model component, the ablation experiments were designed to analyze the effect of different model components. The first ablation experiment is to analyze the effect of Dynamic graph convolutional network. The experimental setup was as follows: models are constructed by using single global graph, single dynamic graph, and different fusion methods of global graph and dynamic graph respectively. The training epoch for all models is 200, the batchsize is 16, and the learning rate is 0.001. The detailed comparison result of models on D-W is illustrated in Table 8.

**Table 8:** Comparison of prediction accuracy with models with different graphs on D-W.

Models	RMSE	MAE	MAPE	R²	MAX
A0(global)	9.5300	7.7743	0.0103	0.9949	30.0125
A1(MA distance)	9.5467	7.6288	0.0102	0.9940	27.9432
A2(trend)	10.2015	7.1690	0.0099	0.9919	31.0079
A0+A1	9.1356	6.7571	0.0093	0.9939	30.7733
A0+A2	7.4745	5.7502	0.0079	0.9950	25.1747
A0+A1+A2	4.3127	3.0215	0.0041	0.9985	13.4692

As shown in Table 8, relying solely on a global adjacency (A0) yields an RMSE of 9.5300, while adopting either Mahalanobis-distance (A1) or trend-based (A2) dynamic graphs in isolation produces similar or even slightly worse errors (9.5467 and 10.2015, respectively). This confirms that a single static or dynamic graph struggles to balance global rhythm and local detail. When the global graph is combined with one dynamic graph, the RMSE drops markedly to 9.1356 (A0 + A1) and 7.4745 (A0 + A2), demonstrating the benefit of joint modeling. Further incorporating both dynamic graphs alongside the global structure (A0 + A1 + A2) achieves the lowest RMSE of 4.3127, the smallest MAE (3.0215), and the highest R² (0.9985). These results validate the proposed multi-graph fusion strategy and highlight that the synergistic use of global and dual dynamic graphs is indispensable for accurate prediction.

The second ablation experimental setup was as follows: each component was systematically removed from the complete model, and the performance of the resulting simplified models was evaluated. The training epoch for all models is 200, the batchsize is 16, and the learning rate is 0.001, 0.001, 0.001, and 0.001, respectively. The results on D-W are represented in Table 9. The detailed breakdown of the prediction results for model on the test dataset D-W is illustrated in Fig11.

**Table 9:** Comparison of prediction accuracy with different models on D-W.

Model	RMSE	MAE	MAPE	R2	MAX
LSTM	11.9857	9.0593	0.0115	0.9905	35.4811
GCN-LSTM	9.5300	7.7743	0.0103	0.9949	30.0115
DYGCN-LSTM	7.5874	6.1877	0.0084	0.9963	25.0434
DYGCN-SLSTM	4.3127	3.0215	0.0041	0.9985	13.4692

In Table 9, the GCN-LSTM outperforms the LSTM by incorporating spatial features between variables. The DYGCN-LSTM possesses the capability to compute an adaptive adjacency matrix in real-time, which provides a superior representation of variable interconnections across different temporal phases compared to a static global adjacency matrix. Consequently, the DYGCN-LSTM exhibits enhanced predictive accuracy relative to the GCN-LSTM. The integration of the SLSTM into the model reinforces its capacity to capture temporal features, resulting in improved and expedited feature extraction. The enhancement of model indicators upon the addition of each component empirically confirms the synergistic contributions of the spatial-temporal modules.

## Conclusion

This paper presents a new deep learning model that merges Dynamic Graph Convolutional Networks with sliding fully‑connected Long Short‑Term Memory networks to predict real‑time temperatures in aluminum smelting furnaces. The model effectively captures dynamic spatial‑temporal relationships among variables, addressing the limitations of traditional methods in handling large numerical variations and dynamic changes within the smelting process. Its ability to capture dynamic spatial‑temporal features makes it well‑suited for this challenging industrial application.

The model is entirely data‑driven, requires no physical priors, and adapts in real time to changing variable correlations, offering strong generalization to other industrial processes. It is readily applicable to non‑ferrous/ferrous metallurgy, chemical reactors, power systems, and smart manufacturing scenarios involving multi‑sensor monitoring under varying operating conditions. However, the model currently faces certain limitations, including high computational complexity that hinders edge deployment, data acquisition latency under real-world operating conditions, and a lack of interpretability. These issues need to be addressed to enhance its practicality. Future work will focus on model lightweighting for edge deployment, while also employing online learning mechanisms to mitigate data latency challenges and meet interpretability requirements, thereby promoting its application validation across a broader range of industrial scenarios.

## Data Availability

The data that support the findings of this study are not openly available due to corporate privacy and are available from the corresponding author upon reasonable request
